# Spatiotemporal characteristics and influencing factors of carbon emissions from civil buildings: Evidence from urban China

**DOI:** 10.1371/journal.pone.0272295

**Published:** 2022-08-04

**Authors:** Jing Wang, Guangjie Du, Mohan Liu

**Affiliations:** Institute of Finance and Economics Research, Shanghai University of Finance and Economics, Shanghai, China; Institute for Advanced Sustainability Studies, GERMANY

## Abstract

Carbon emissions from civil buildings refer to the carbon emissions generated during the operation of civil buildings. With the continuous development of the urban economy and the improvement of people’s living standards, this part of carbon emission puts tremendous pressure on China to achieve the goal of carbon peaking and carbon neutral. In the context of rapid urbanization, studying the spatiotemporal characteristics and influencing factors of the carbon emissions from civil buildings have strong practical significance for China to achieve the "dual carbon" goal. Based on the emission data from 104 prefecture-level cities in China, we examine the spatiotemporal characteristics of the civil building carbon emissions from the perspectives of temporal evolution trend, spatial distribution and its dynamic evolution, spatial difference and its decomposition, and spatial autocorrelation characteristics. Finally, we reveal the influencing factors of the carbon emissions from civil buildings using static panel data models and spatial dynamic panel data models. The results of the study show that: (1) During the sample period, the carbon emissions from civil buildings have increased year by year. The civil building carbon emissions have become an important source of China’s overall carbon emissions. Realizing energy saving and emission reduction in the operational stage of civil buildings is crucial to realizing China’s "dual carbon" goal. (2) According to the estimated results, there is a significant inverted U-shaped non-linear relationship between urbanization and civil building carbon emissions. Most Chinese cities are located in the upward part of the inverted U-shaped curve at present. Thus, the traditional economic growth model characterized by high energy consumption and high emission during rapid urbanization should be abandoned to reduce the carbon emissions from civil buildings. (3) Technological progress and fixed asset investment can effectively reduce the carbon emissions from civil buildings. At the same time, the level of marketization and social consumption expenditure positively affect the carbon emissions from civil buildings. It is necessary to improve the relevant market mechanisms, policy subsidies, and other means to encourage the application of green energy-saving technologies in civil buildings. Also, it is needed to guide the urban residents’ consumption structure and lifestyle in a low-carbon direction, to reduce the energy consumption and carbon emissions during the operation of civil buildings.

## 1 Introduction

Since the reform and opening up, China has experienced the largest and fastest urbanization in the history of the world. The proportion of urban population in China has increased from 20.6% (1982) to 63.89% (2020), while the rapid expansion of cities has brought about a rapid increase in energy consumption and carbon dioxide emissions which causes a negative impact on the climate system and ecological environment. China has become the world’s largest emitter of carbon dioxide, and its total carbon emission in 2020 was about 10 billion tons, accounting for 29% of the global carbon emissions. China’s "dual carbon" goal aims to propel the economy onto a high-quality development path through carbon emission commitments. Against this background, a comprehensive understanding of the relationship between urbanization and carbon emissions is crucial to formulating future carbon emission reduction policies and achieving carbon neutrality goals in China.

Regarding the primary sources of total carbon emissions during urbanization, many academic studies have been conducted on this issue [1–6]. Scholars have analyzed the impact of urbanization on carbon emissions from different perspectives, such as industrial production [7, 8], construction industry [9], transportation [10], and urban residential energy consumption. Among them, carbon emissions from buildings are regarded as an important source of overall carbon emissions, and building carbon emissions can reach 27.9% - 34.3% of China’s total carbon emissions [11]. So exploring the impact of urbanization on building carbon emissions not only helps to alleviate the pressure of carbon emission reduction caused by the rapid growth of urban construction, but is also beneficial to achieving China’s "dual carbon" goal. In the study on the relationship between urbanization and building carbon emissions, the existing literature mainly analyzes the issue from the perspective of the construction industry. The existing literature mainly focuses on the increase of carbon emissions caused by the construction process of buildings, while ignoring the carbon emissions generated by energy consumption during the operation of buildings. Since buildings usually have a long life cycle, the carbon emissions generated during the operation of buildings generally account for 75% of their whole life cycle carbon emissions. If the energy consumption generated during the operation of buildings is ignored, the impact of the building sector on the total carbon emissions will not be fully understood, thus reducing the effectiveness of relevant carbon reduction policies.

In order to remedy the above deficiencies of existing studies, we take the carbon emissions from civil buildings as the research object, and examine the carbon emissions generated by energy consumption during the operation of buildings related to the tertiary industry and residential energy consumption. Firstly, based on the concepts of energy consumption and carbon emissions from civil buildings, we account for the information related to carbon emissions from civil buildings in 104 prefecture-level Chinese cities from 2010 to 2018. Secondly, we examine the spatiotemporal characteristics of the civil building carbon emissions from the perspectives of temporal evolution trend, spatial distribution and its dynamic evolution, spatial difference and its decomposition, and spatial autocorrelation characteristics. Finally, the theoretical analysis is constructed by combining the improved *STIPRAT* model with the *EKC* hypothesis. Empirical methods such as static panel data models and spatial dynamic panel data models are used to examine the influencing factors of carbon emissions from civil buildings. This study helps to expand the understanding of the relationship between urban development and carbon emissions from civil buildings, and thus provides a scientific basis and decision support for formulating more reasonable carbon emission reduction policies under the background of China’s continuous urbanization process.

## 2 Literature review

As ecological and environmental issues such as climate warming have increasingly become considerable challenges in sustainable social and economic development, how to reduce carbon dioxide and other greenhouse gases has become the focus of attention in the academic community. Carbon emissions are mainly generated in urban areas where population, buildings, and socio-economic activities are concentrated. With the rapid urbanization since the reform and opening-up, China’s urban energy consumption and carbon emissions have increased significantly, making China face severe pressure to reduce carbon emissions. Liddle et al. [12] found that urbanization has a significant positive effect on energy consumption and carbon emissions based on data from a sample of 17 developed countries. Lin & Liu [13] used time-series data on carbon emissions and other socio-economic variables to examine the influencing factors of carbon emissions in China. The study found that adding the urbanization variable can more accurately identify the long-term equilibrium relationship between various key variables and carbon emissions. In addition, scholars such as Zhang & Lin [14] and Liu & Bae [5] have also conducted many studies on the relationship between urbanization and carbon emissions in China. Most existing studies show that urbanization has a significant impact on carbon emissions. However, there are still different opinions on the direction of the relationship between urbanization and carbon emissions [15–17]. For example, Zhang & Lin [14] proposed that there is a significant positive relationship between urbanization and carbon emissions. The development of urbanization will bring more energy consumption while generating more carbon emissions. Fan et al. [18] and Wang et al. [19] investigated the negative effect mechanism between urbanization and carbon emissions. They propose that carbon emissions can be reduced due to the technological progress and infrastructure improvement brought about by urbanization. Xu & Lin [20] examined the dynamic impact of China’s urbanization on carbon emissions based on time series data and provincial panel data. The study shows that there is an inverted U-shaped Kuznets curve relationship between the process of urbanization and carbon emissions. In addition, some scholars have pointed out that the relationship between urbanization and carbon emissions is uncertain, with its specific direction and intensity being influenced by economic development and other regional characteristics [21]. Huo et al. [22] showed that the impact of urbanization on carbon emissions varies with different stages of income and energy structure according to the empirical results from panel threshold regression models. They pointed out that the driving effect of urbanization on carbon emissions presents a "stepwise growth" when a certain threshold is exceeded.

During the rapid process of urbanization, the traditional socio-economic relations and industrial structure will inevitably undergo profound changes, which will significantly impact on the total carbon emissions. In this process, the carbon emissions of different industry sectors will show different characteristics due to different resource endowment conditions, production factor requirements, technological levels, and development stages. Many fruitful studies have been conducted to analyze carbon emissions in different fields or sectors during urbanization, such as industrial carbon emissions [23, 24], agricultural carbon emissions [25, 26], and carbon emissions from service industry [27, 28]. Along with the rise of urbanization, the development of the construction industry will be accompanied by increasing energy consumption and carbon emissions. Existing studies have investigated the spatial distribution and influencing factors of carbon emissions in the building sector [29–31]. The carbon emissions from the construction sector include carbon emissions generated during the construction of buildings and the installation of related equipment. According to Hong et al. [32], this part of carbon emissions already accounts for one-third of the total carbon emissions in China. Since buildings usually have a long life cycle, the carbon emissions caused by buildings are not only generated during the construction process, but also during their whole life cycle due to the energy consumption during operation, which is an important source of carbon emissions. Some scholars have studied the relationship between urbanization and building carbon emissions during the operation stage [33, 34]. Although these papers have provided rich information on the relationship between urbanization and building carbon emissions, there are still some limitations in the existing studies: (1) the existing literature mainly focuses on building carbon emissions from secondary industry, while ignoring carbon emissions from civil buildings; (2) the existing studies have not reached a consensus on the relationship between urbanization and building carbon emissions, and the conclusions are quite different; (3) the heterogeneous characteristics of the evolution of building carbon emissions in the process of urbanization are failed to reveal clearly.

The energy consumption of civil buildings accounts for 22%-25% of China’s total power generation, which puts tremendous pressure on China’s energy industry and carbon emission reduction. In the current literature, the carbon emissions of a single type of civil building or a single building during its life cycle have been analyzed using life cycle assessment methods and IPCC inventory methods. However, fewer studies have been conducted on civil building energy consumption at the city level, especially in terms of revealing the characteristics of civil building energy consumption. In addition, the impact of urbanization on civil building energy consumption and carbon emissions has not been fully revealed. In response to the shortcomings mentioned above, we will take 104 prefecture-level cities in China as the research sample to calculate the carbon emissions generated by civil buildings from 2010 to 2018. Then we examine the spatial pattern and evolution trend of China’s civil building carbon emissions. The influencing factors of civil building carbon emissions are finally revealed to provide a reference for formulating more targeted carbon reduction policies.

## 3 Model and data

### 3.1 Model specification

We use the improved *STIRPAT* model and combine it with the *EKC* hypothesis to construct the basic theoretical analysis framework. Ehrlich & Holdren [35] proposed the *IPAT* equation to reflect the impact of population and resource consumption on ecosystems. The *IPAT* Equation is *I* = *P*×*A*×*T*, where *I* represents the impact on ecosystems, *P* represents the population size, *A* represents the economic factor, and *T* represents the technology. The multiplication sign in the above equation only indicates that there is a specific relationship between population size, economic factors, and technology. The *IPAT* equation, although succinctly representing the relationship between the impact on ecosystems and their influencing factors, is not flexible for economic analysis. York et al. [36] proposed the *STIRPAT* model to solve the above problem. The equation of the *STIRPAT* model is shown in Eq (1), where *a*, *b*, *c*, and *d* are parameters to be estimated. *e* is a random error term.


$$I=aP^{b}\times A^{c}\times T^{d}e$$ (1)

Based on the above *STIRPAT* model, it is possible to estimate the impact of each factor on the environment by regression models. However, Eq (1) expresses only the linear relationship among variables. According to the EKC hypothesis, there is an inverted U-shaped non-linear relationship rather than a simple linear relationship. Based on this view, the *STIRPAT* model can be improved by including diversified variables (such as per capita income and urbanization) and their squared terms, so that the influence factors of resource consumption or carbon emissions can be examined more comprehensively. We constructed the following econometric model based on the improved *STIRPAT* model, as shown in Eq (2).


$$\begin{array}{c} LnBCO_{2it}=\alpha_{it}+\beta_{1}LnCO_{2it-1}+\beta_{2}LnPop_{it}+\beta_{3}LnA_{it}+\beta_{4}{\left(LnA_{it}\right)}^{2}+\beta_{5}LnURB_{it}\\ +\beta_{6}{\left(LnURB_{it}\right)}^{2}+\beta_{7}LnEff_{it}+\sum_{k=1}^{K}X_{it}^{k}+e_{it} \end{array}$$ (2)

Specifically, Eq (2) expresses the carbon emissions from civil buildings in terms of *BCO*_*2it*_, where the subscripts *i* and *t* characterize the observation sample and time, respectively. The explanatory variables include: (1) The core explanatory variables mainly include urbanization(*URB*_*it*_) and the squared term of urbanization. In this way, we can study whether the impact of urbanization on civil building carbon emissions shows a non-linear trend. (2) In addition, considering the requirements of variable setting in the *STIRPAT* model, the population size of each city is used to characterize the demographic factors (*Pop*). The per capita disposable income of urban residents (*Inc*) and the total retail sales of consumer goods (*Con*) represent economic factor *A* from social income and expenditure perspectives, respectively. The technical level *T* is characterized by energy efficiency (the ratio of the added value of the tertiary industry to the total energy consumption of civil buildings, *Eff*) and the number of patents granted per 10,000 people in each city (*Tech*). (3) Considering that the factors influencing civil building carbon emissions are much more complicated than the general resource and environment system, there may be a problem with omitted variables if only the above variables are included in the model. Therefore, we add a series of control variables (*X*_*it*_) to the *STIRPAT* model, including industrial structure (measured by the proportion of tertiary industry to GDP, *Str*) and market development level (*Mar*).

### 3.2 Data

Broadly speaking, the scope of civil building energy consumption involves the secondary industry and civil life, including the energy consumption generated during the construction of the building and the entire life cycle of the building, that is, the energy consumption of the building industry and the energy consumption of the building during operation. The energy consumption of civil buildings in a narrow sense focuses on the building operation phase, and concentrates on the energy consumption generated by providing services to users in residential and recreational industry buildings, mainly including energy consumption for the operation of residential buildings and energy consumption for the operation of tertiary industry buildings. According to the above concepts, we select civil building energy consumption in the narrow sense as the research object: first, civil building usually has a long life cycle, and the energy consumption generated during its entire operation phase is large; second, civil building energy consumption in the broad sense covers industrial production processes such as building material production and building construction. The expansion of cities during urbanization will drive the development of the construction industry to a large extent. So this part of energy consumption is bound to increase significantly during rapid urbanization. Therefore, the exclusion of energy consumption in the construction sector can concentrate more on the energy consumption generated during the operation of civil buildings.

The energy consumption during the operation of civil buildings does not only refer to the energy consumption generated by civil buildings themselves, but also includes a wider range of energy consumption during the operation of civil buildings, mainly related to heating, air conditioning, office equipment, hot water supply, household appliances. However, there is a lack of authoritative statistics on the energy consumption of civil buildings in comprehensive spatial scope. In this paper, we will use the existing data in the statistical yearbooks to obtain the estimated civil building energy consumption data. The remaining part of the total energy consumption of the tertiary industry minus the energy consumption of the transportation, storage, and postal industries can be used as an indicator for energy consumption from the operation of tertiary industry building. The residential energy consumption of urban and rural residents can be used as an indicator for energy consumption during the operation of residential buildings. Then, by summing up the above two parts of energy consumption, the estimated energy consumption of civil buildings can be obtained. Subject to the degree of data disclosure, we identified 104 prefecture-level cities (including 49 eastern cities, 39 central cities, and 16 western cities) as the study sample, spanning the period from 2010 to 2018.

According to the functional classification of buildings, the energy consumption of civil buildings can be divided into energy consumption during the operation of tertiary industry buildings and energy consumption during the operation of residential buildings. According to the *National Economic Classification of Industries* formulated by the National Bureau of Statistics of China, the energy consumption during the operation of tertiary industry buildings includes the total energy consumption of "information transmission, computer services, and software industry", "commerce, accommodation, and catering industry", "finance, real estate, business, and residential services" and other industries. The energy consumption of "transportation, storage, and postal industry" is mainly transportation energy consumption, which is not included in the accounting of civil building energy consumption. The energy consumption during the operation of residential buildings is determined as " urban and rural residential energy consumption." Given that the energy consumption of civil buildings is mainly used for building heating, cooling, hot water supply, cooking, lighting, and household appliances, the energy consumption of civil buildings is obtained by summing up the following five components: electricity consumption in specific industries and residential sectors, total central heating for steam and hot water, artificial gas, natural gas, and liquefied petroleum gas for residential households.

Based on the above data, we will further use the carbon emission estimation method provided by IPCC to account for the carbon emissions from civil buildings in 104 Chinese cities, as shown in the following formula.


$${\mathrm{TBCO}}_{2}=\sum_{i}^{n}E_{i}\times P_{i}\times e_{i}\times \frac{44}{12}$$ (3)

In Eq (3): *TBCO*_*2*_ refers to the carbon emissions from civil buildings, *E*_*i*_ represents the consumption of energy type *i*, *P*_*i*_ denotes the carbon emission factor of energy type *I*, while *e*_*i*_ denotes the standard coal conversion factor of energy type *i*. *n* is the number of energy types, and 44/12 is the ratio of the molecular weight of carbon dioxide to carbon.

## 4 Results and analysis

### 4.1 Spatiotemporal characteristics of carbon emissions from civil buildings

#### (1) Temporal trend of carbon emissions from civil buildings

**Fig 1:**
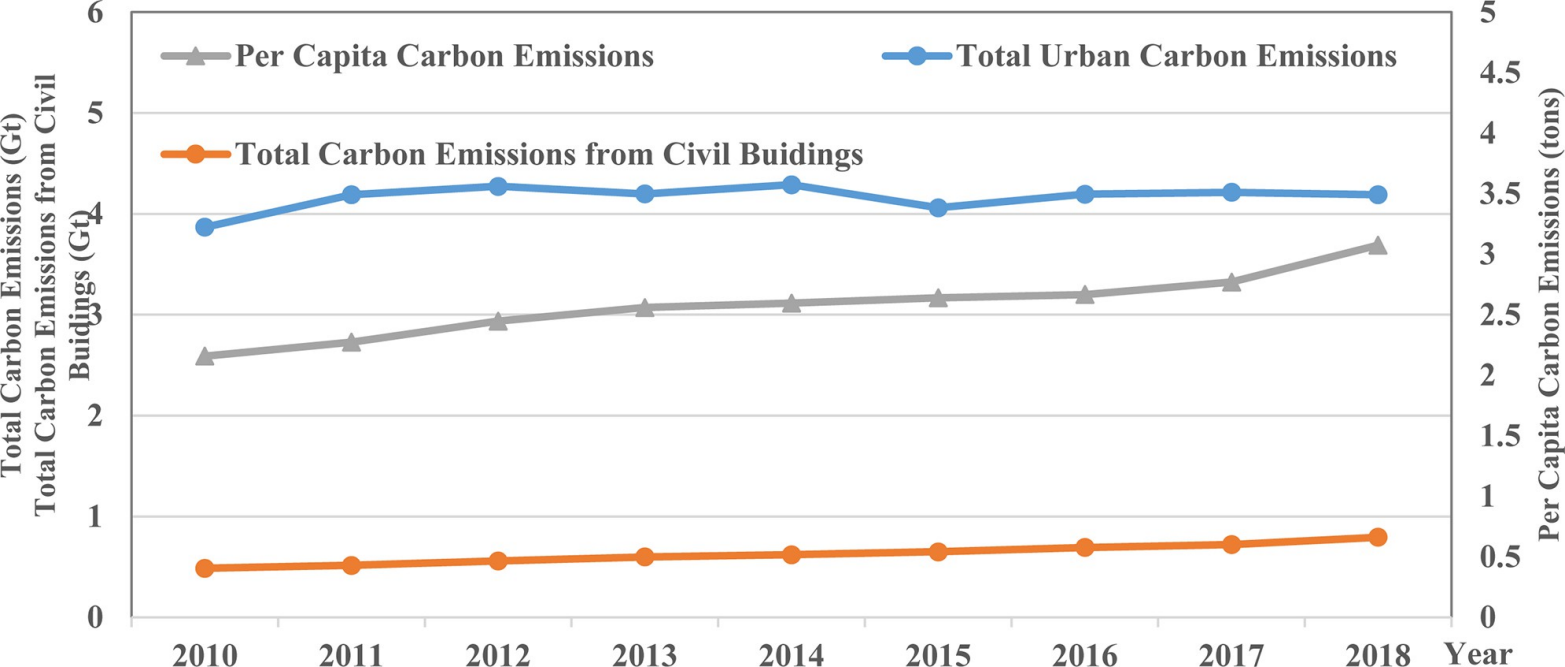
The temporal trend of carbon emissions from civil buildings in China.

With the rapid development of urbanization, China’s urban population continues to grow, and the carbon emissions from urban civil buildings are also increasing year by year. Fig 1 shows the temporal trend of carbon emissions from urban civil buildings in China. The total carbon emissions from civil buildings in 104 sample cities increased from 0.48 Gt in 2010 to 0.79 Gt in 2018 (1 Gt = 10^9^ tons), with an annual growth rate of 6.30%; while the per capita carbon emissions from civil buildings grew from 2.15 tons/person to 3.07 tons/person, with an annual growth rate of 4.53%. Further analysis can find that the total carbon emissions of civil buildings increased relatively flat during the sample period, and the per capita carbon emissions of civil buildings increased rapidly during 2010–2012 and 2017–2018 while insignificantly changing in other periods. In addition, Fig 1 reports the temporal trend of total urban carbon emissions in China during the sample period. In 2010, the carbon emissions from civil buildings accounted for 12.6% of the total urban carbon emissions, while the percentage increased to 20% in 2018. The above results indicate that the carbon emissions from civil buildings have become an important source of the overall urban carbon emissions.

**Fig 2:**
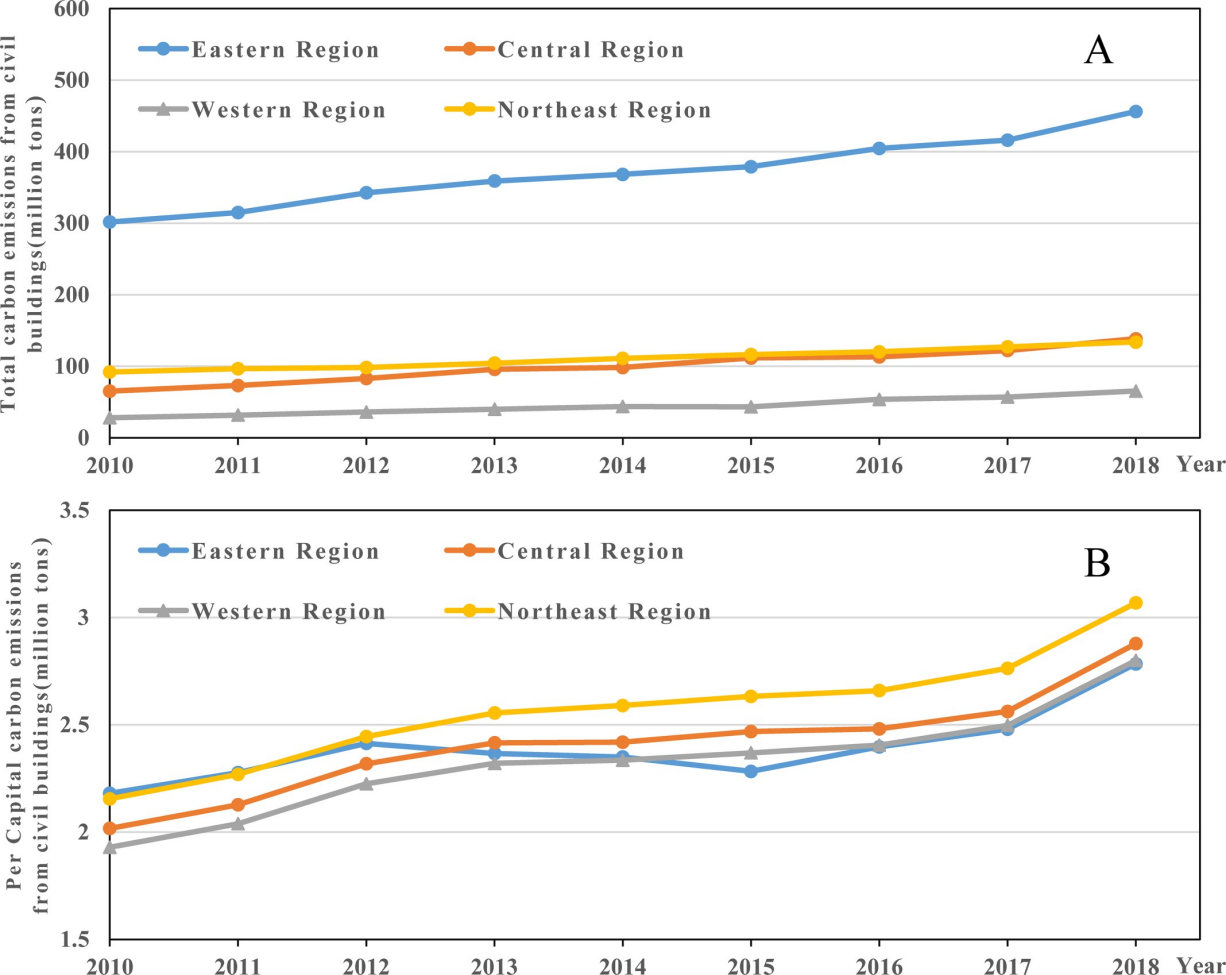
The temporal trend of total carbon emissions from civil buildings (A) and per capita carbon emissions from civil buildings (B) in four major Chinese regions.

We further examine the temporal trend of carbon emissions from civil buildings in four major Chinese regions (Fig 2). As seen in Fig 2, the total carbon emissions from civil buildings in the eastern region were significantly higher than in other regions. The total civil building carbon emissions in the eastern region were 301.68 million tons in 2010 and roused to 456.18 million tons in 2018, with an annual growth rate of 5.30%. The total carbon emissions of civil buildings in the central and western regions experience relatively rapid growth during the sample period, with annual growth rates of 9.87% and 11.19%, respectively. The annual growth rate of total carbon emissions from civil buildings in the Northeast is 4.80%. Regarding the per capita civil building carbon emissions, there was a certain degree of divergence among the four major regions. Among them, the northeast region was in the first tier, with per capita values rising from 2.15 tons per person in 2010 to 3.07 tons per person in 2018. The central and western regions were located in the second tier. The eastern region was in the first tier at the beginning of the sample period; however, the growth trend of per capita values slowed down after 2014. The eastern region was in the second tier by the end of the sample period.

**Fig 3:**
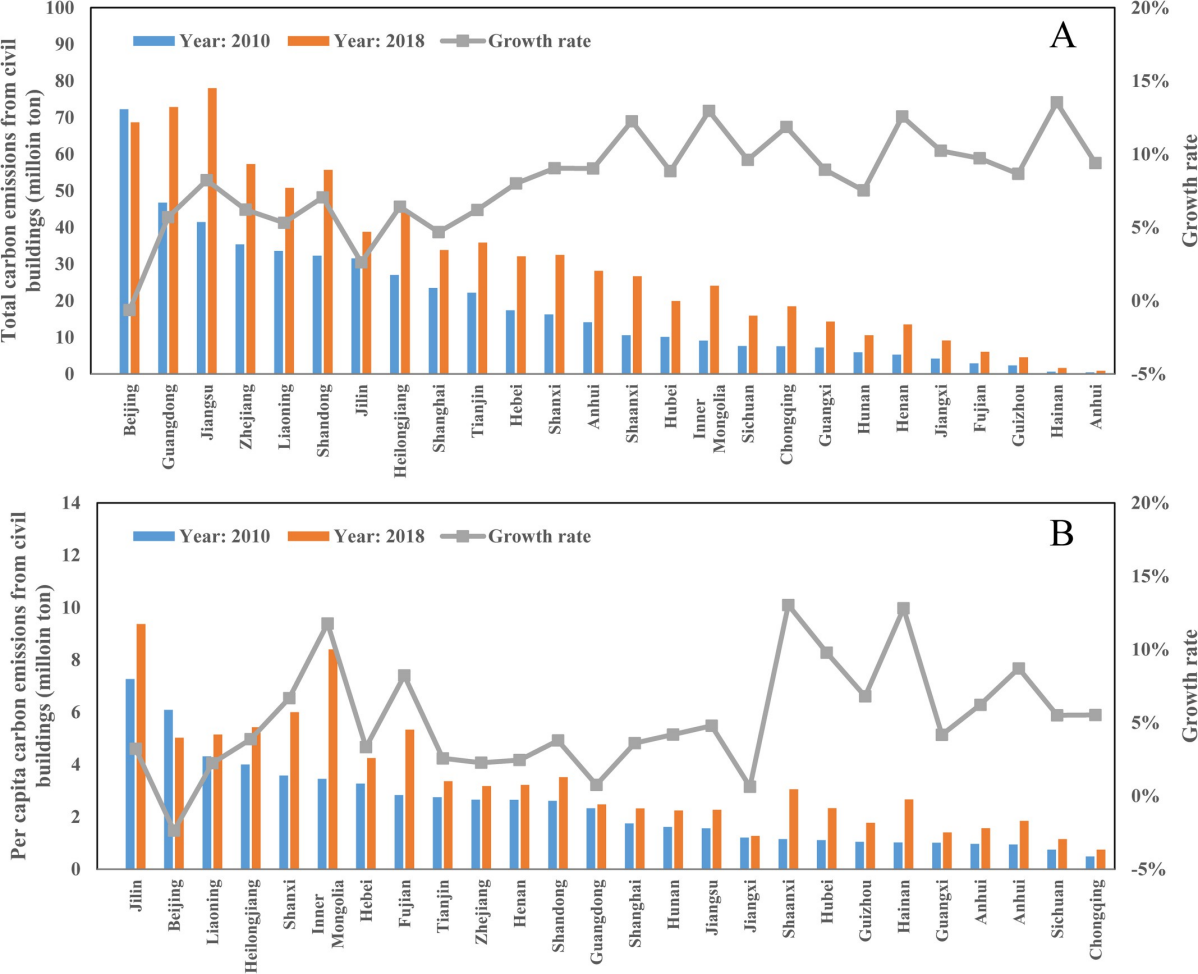
The temporal trend of total carbon emissions from civil buildings (A) and per capita carbon emissions from civil buildings (B) in Chinese provinces.

From the provincial perspective (Fig 3), the total carbon emissions from civil buildings in Hainan, Inner Mongolia, Henan, Shaanxi, Chongqing, and Jiangxi increased significantly during the sample period. Among them, the total carbon emissions of urban civil buildings in Hainan Province increased the most, with an average annual growth rate exceeding 13.53%. In contrast, the total values in Guangdong, Liaoning, Shanghai, Jilin, and Beijing increased slightly (e.g., the growth rate in Beijing was -0.63%). In addition, the per capita carbon emissions of civil buildings in Shaanxi, Hainan, and Inner Mongolia grew substantially (the increase in Shaanxi Province is 13.03%). On the other hand, Guangdong, Jiangxi, and Beijing had a slight increase in per capita values. The per capita civil building carbon emissions in Beijing decreased from 6.08 tons/person in 2010 to 5.02 tons/person in 2018.

#### (2) Spatial distribution of carbon emissions from civil buildings

**Fig 4:**
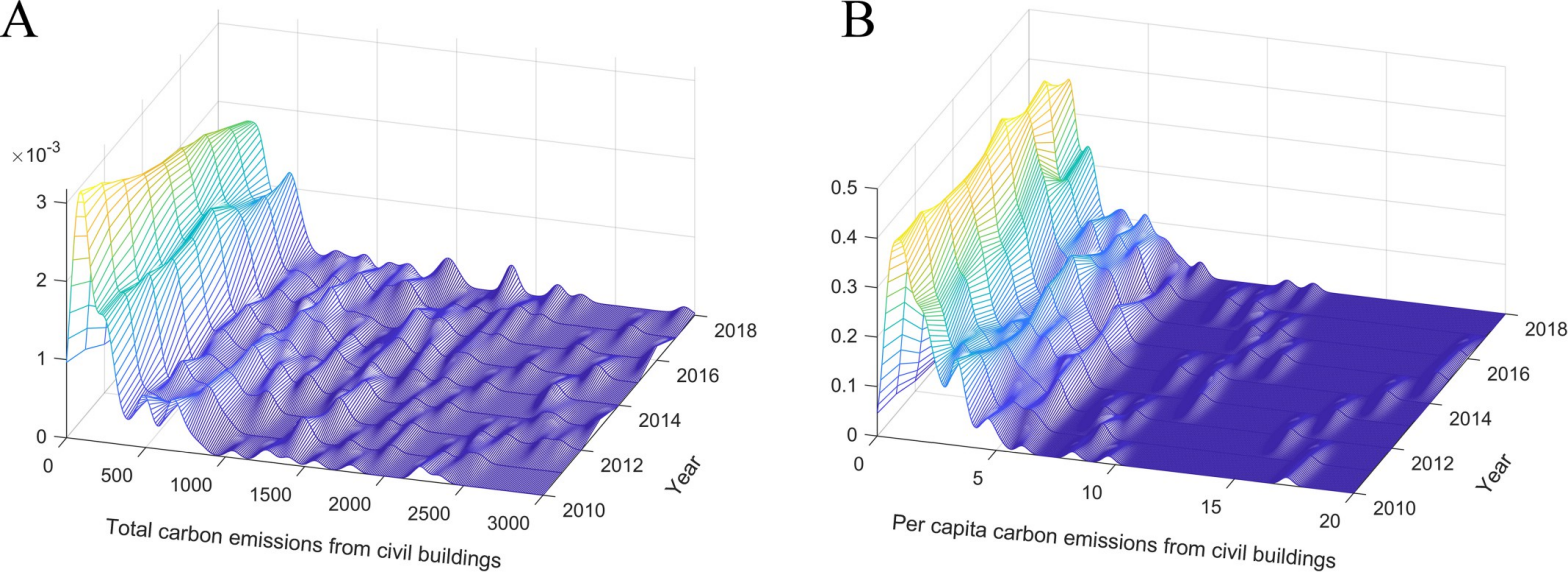
Kernel density curve of total carbon emissions from urban civil buildings (A) and per capita carbon emissions from civil buildings (B).

Based on the kernel density estimation method [37, 38], Fig 4 depicts the spatial distribution of the total carbon emissions and per capita emissions from civil buildings in Chinese cities. As a non-parametric method, kernel density estimation can describe the distribution pattern of random variables through a continuous density curve to reflect the variable distribution’s location and shape. According to Fig 4, the kernel density curve of the total carbon emissions from civil buildings shows a significant multi-peak distribution. In addition to the main peak near the sample mean, the kernel density curve has multiple high-level secondary peaks and a longer right tail. The above results show that the distribution of total carbon emissions from urban civil buildings has a strong characteristic of spatial differences. The civil building carbon emissions in a small group of cities are much higher than in other sample cities. During the sample period, the main peak of the kernel density curve gradually shifts to the right, and the peak value gradually decreases, indicating that the spatial difference in total carbon emissions from civil buildings gradually decreased over time. Compared with 2010, the right-tailing of the kernel density curve did not change significantly in 2018, indicating that the total carbon emissions from civil buildings in a small group of cities are higher than in other regions. From the kernel density estimation results of per capita civil building carbon emissions, the kernel density curve presents the distribution pattern of multiple peaks. The peak value of the main peak is much higher than the secondary peaks, and the positions of the secondary peaks are closer to the main peak. Additionally, the kernel density curve does not form an obvious right tail. The spatial difference in per capita values is relatively small compared to the total values. Most cities are concentrated near the sample average, and cities with higher per capita values have not widened the gap with other cities.

**Fig 5:**
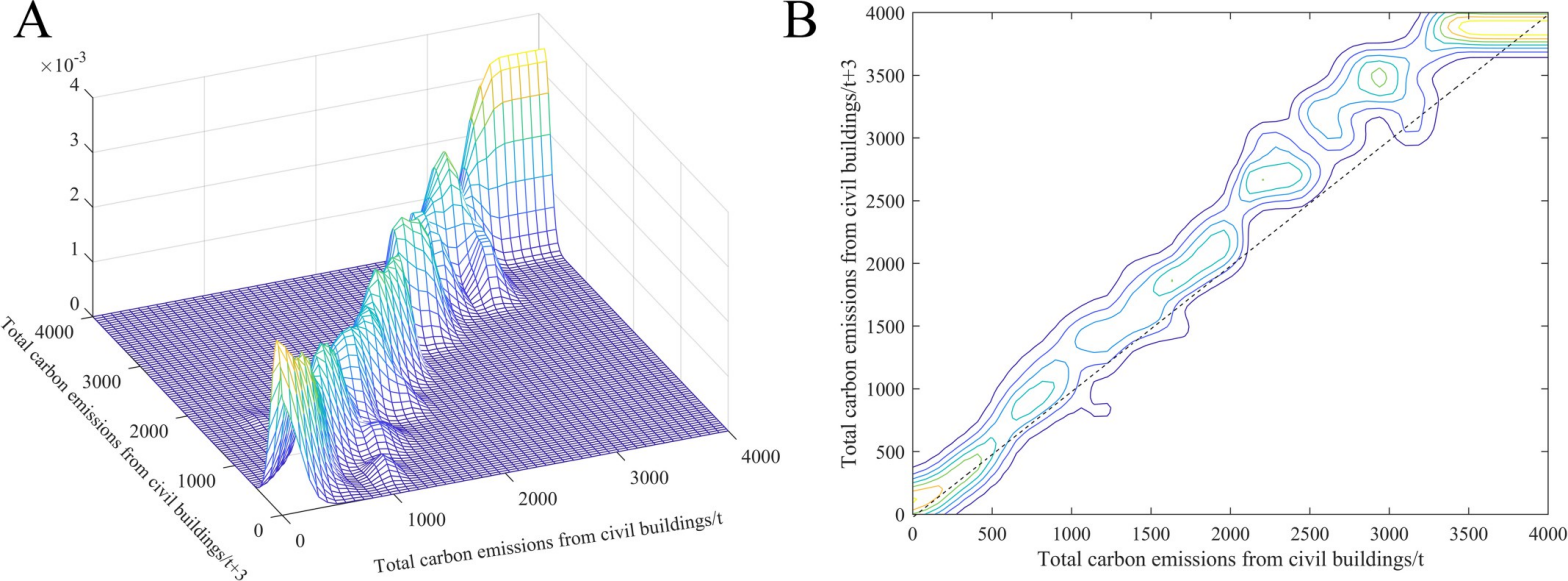
Dynamic stochastic kernel density curve (A) and density contour (B) of total carbon emissions from civil buildings.

Traditional kernel density estimation can depict the static distribution of carbon emissions from civil buildings. By comparing the kernel density curves in different years, we can roughly see how the spatial distribution has changed during the sample period. However, the traditional kernel density estimation can only provide limited information on the dynamic evolution of spatial distribution. Stochastic kernel density estimation can reveal the spatial distribution pattern and dynamic evolution trend of carbon emissions from civil buildings by analyzing the probability density functions of state transitions under various conditions. Based on the stochastic kernel density estimation method, we examine the transition probability of carbon emissions from civil buildings under dynamic condition. Fig 5 shows the dynamic stochastic kernel density curve of the total carbon emissions from civil buildings and the corresponding density contours. Generally, the dynamic stochastic kernel density curve can be divided into three ranges. When the carbon emissions of civil buildings are less than 6 million tons, the kernel density curve is mainly distributed on both sides of the 45° diagonal; the carbon emissions from civil buildings in this interval remain roughly unchanged. When the emissions are higher than 6 million tons, the kernel density curve gradually deviates from the diagonal, indicating that the carbon emissions from civil buildings show a certain growth trend. When the emissions are higher than 35 million tons, the kernel density curve is parallel to the x-axis, which shows that the carbon emissions of civil buildings in this interval have a convergence trend. In summary, the total carbon emissions from civil buildings in Chinese cities are showing a steady growth trend, and the relative gap between cities is gradually decreasing.

**Fig 6:**
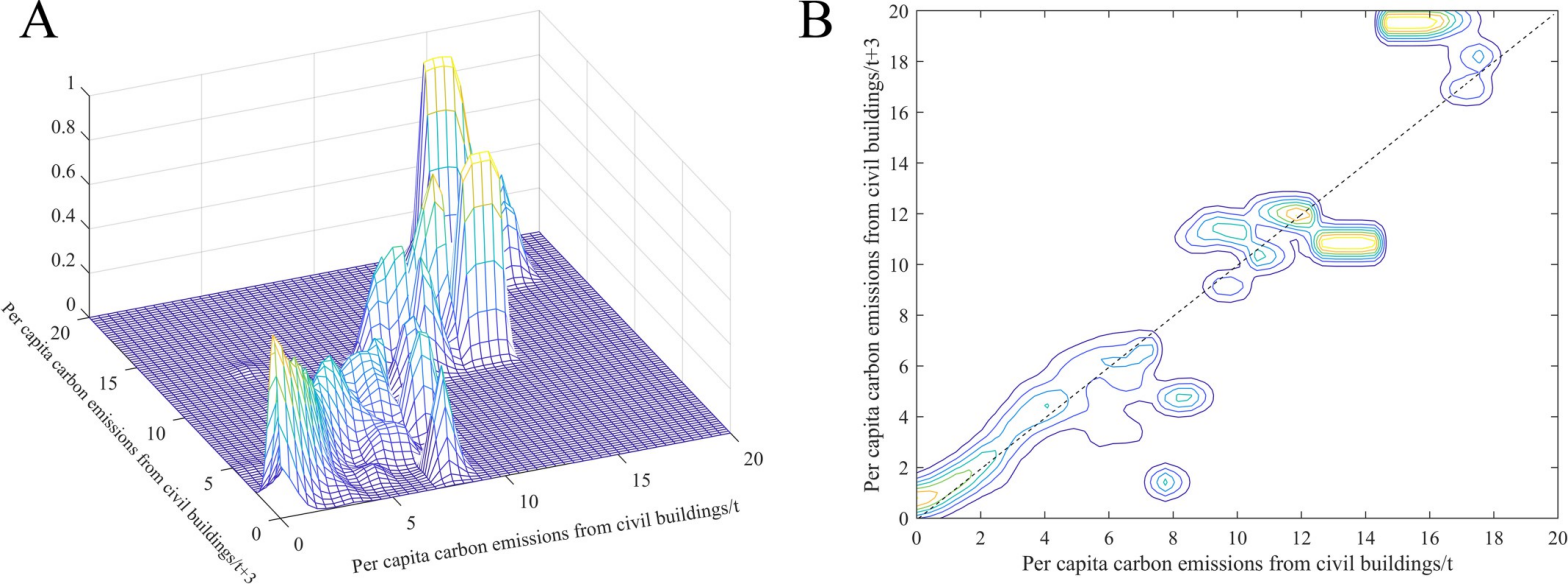
Dynamic stochastic kernel density curve (A) and density contour (B) of per capita carbon emissions from civil buildings.

Fig 6 shows the dynamic stochastic kernel density curve of per capita carbon emissions from civil buildings and the corresponding density contour. It can be seen that the kernel density curve of the per capita values can be divided into two interval ranges. When the per capita values are lower than 10 tons/person, the dynamic stochastic kernel density curve is mainly concentrated near the 45° diagonal. When the per capita values are higher than 10 tons/person, the kernel density curve is mainly parallel to the x-axis. The per capita civil building carbon emissions show a convergence trend. The above results show that compared to year *t*, the spatial distribution of per capita civil building carbon emissions in year *t+*3 only has a minor change, while the high emission cities show a convergence trend.

#### (3) Spatial differences in carbon emissions from civil buildings

To further characterize the spatial differences in carbon emissions from civil buildings in China, we measure the spatial differences in total carbon emissions from civil buildings and per capita carbon emissions from civil buildings according to the Gini coefficient (Table 1). Moreover, the Gini coefficient is decomposed according to China’s four major regions based on the decomposition method proposed by Dagum [39].

**Table 1:** The result of the Gini coefficient and its decomposition.

Year	Total.				Per capita.			
	Gini	Within	Between	Trans.	Gini	Within	Between	Trans.
2010	0.629	0.218	0.328	0.083	0.404	0.146	0.135	0.122
2011	0.626	0.217	0.317	0.092	0.407	0.150	0.127	0.130
2012	0.609	0.213	0.302	0.094	0.385	0.141	0.108	0.136
2013	0.601	0.210	0.288	0.103	0.385	0.140	0.099	0.146
2014	0.605	0.209	0.290	0.106	0.399	0.143	0.103	0.153
2015	0.608	0.211	0.278	0.118	0.408	0.145	0.128	0.136
2016	0.596	0.205	0.277	0.114	0.376	0.135	0.097	0.144
2017	0.585	0.198	0.272	0.115	0.366	0.130	0.092	0.145
2018	0.572	0.193	0.260	0.118	0.364	0.130	0.088	0.146

According to the Gini coefficients, the total carbon emissions from civil buildings in China exhibit significant spatial non-equilibrium characteristics. From 2010 to 2013, the overall Gini coefficients showed a gradual downward trend, with an average annual decrease of 1.51%. Since then, the Gini coefficient has remained around 0.6. Furthermore, the downward trend of the overall Gini coefficient has accelerated since 2016, with an average annual decrease of about 2.02%. By decomposing the overall Gini coefficient, the spatial differences of total carbon emissions from urban civil buildings can be decomposed into spatial differences within sub-regions, spatial differences between sub-regions, and the intensity of transvariation.

**Fig 7:**
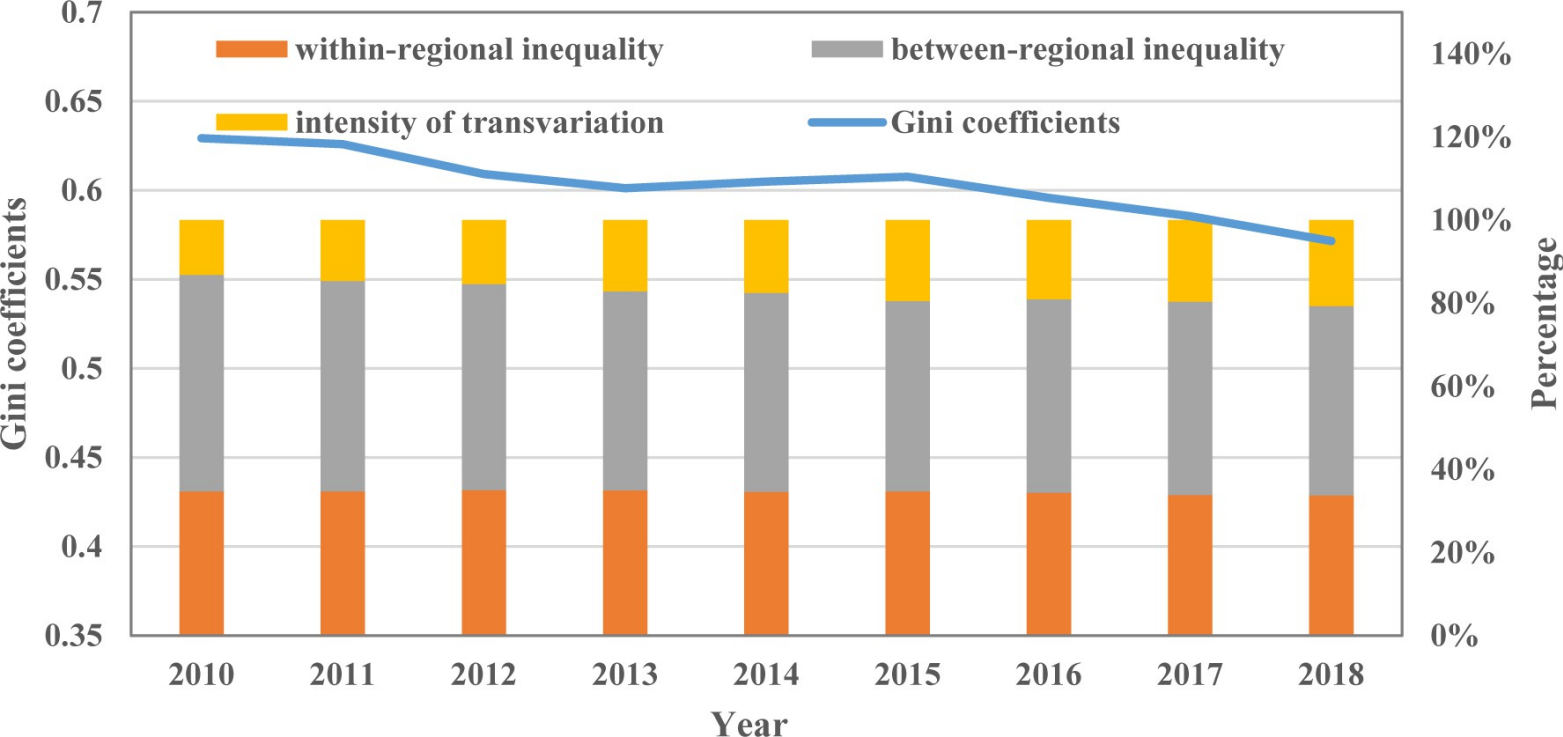
The spatial difference in total carbon emissions from civil buildings.

According to the decomposition of Gini coefficients in Fig 7, about 34.5% of the overall spatial difference comes from the inequality within regions. In comparison, the average contribution of inequality between regions and the intensity of transvariation is 48.1% and 17.4%, respectively. It can be seen that the differences between regions have become the main source of spatial differences in the total carbon emissions from civil buildings in China. During the sample period, the contribution of within-regional inequality remained roughly unchanged. The contribution of between-regional inequality decreased from 52.1% in 2010 to 45.6% in 2018, while the contribution of intensity of transvariation increased from 13.2% in 2010 to 20.7% in 2018. This phenomenon indicates that the degree of crossover and overlap in the spatial distribution of total carbon emissions from civil buildings has increased during the sample period. The hierarchical spatial distribution characteristics are gradually weakened.

**Fig 8:**
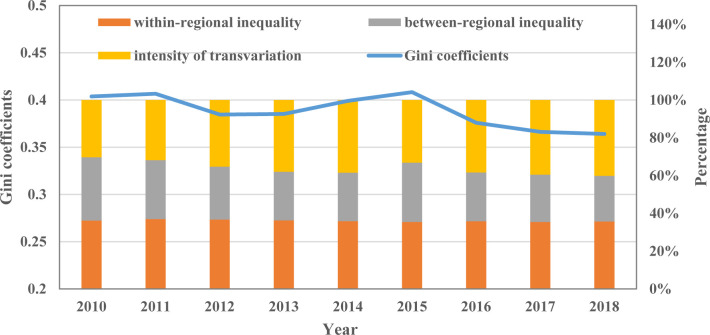
The spatial difference in per capita carbon emissions from civil buildings.

According to the Gini coefficients of per capita carbon emissions from civil buildings (Fig 8), the spatial differences of per capita carbon emissions from civil buildings show fluctuating changes. From 2000 to 2015, the Gini coefficients showed a trend of decreasing first and then increasing. The average value of Gini coefficients during this period was 0.40. After that, the Gini coefficients showed a downward trend, and the Gini coefficients dropped to 0.36 in 2018, with an average annual decrease of 3.75%. From the decomposition of the Gini coefficients, within-regional inequality was the main source of overall spatial differences at the beginning of the sample period, with a contribution rate of about 36.2%. Since then, within-regional inequality has remained unchanged, and its share in the overall spatial differences has remained at about 36%. Between-regional inequality decreases significantly during the sample period, while the contribution of the intensity of transvariation gradually increases. In 2018, the contribution of intensity of transvariation was 40.1%, which became the main source of overall spatial differences. The above phenomenon indicates that the previous hierarchical spatial distribution characteristics have also been broken at the per capita level, and the crossover phenomenon between regions has been strengthened. Based on the emission data, it can be found that the reason for the above phenomenon is that the per capita carbon emissions from civil buildings in the eastern cities have dropped from the original first tier to the second tier, while the per capita carbon emissions from civil buildings in the central and western regions are gradually approaching each other.

#### (4) Spatial autocorrelation of carbon emissions from civil buildings

The Gini coefficients show that the carbon emissions from civil buildings in China have significant spatial non-equilibrium characteristics. We will conduct spatial autocorrelation analysis on this non-equilibrium characteristic to examine whether it shows a random distribution pattern. The Moran index in spatial statistics is one of the standard indexes to measure spatial autocorrelation [40], and the calculation formula is shown in Formula (4). *N* is the number of sample cities, *X*_*i*_ denotes the carbon emissions from civil buildings in city *i*, and $\bar{X}$ is the sample mean. *W(i*, *j)* denotes the spatial weight between city *i* and city *j*. If the Moran index, which takes the value in [–1, 1], is greater than zero, it indicates that there is a positive spatial autocorrelation, and the civil building carbon emissions present an agglomeration pattern of high—high values and low—low values. If the Moran index is less than zero, it means that there exists negative spatial autocorrelation; the civil building carbon emissions present a discrete distribution pattern, that is, a cluster of high values and low values. A zero value indicates that the spatial difference is in a random distribution pattern.


$$I=\frac{N}{S_{0}}\frac{\sum_{i=1}^{N}\sum_{j=1}^{N}W(i,\;j)(X_{i}-\bar{X})(X_{j}-\bar{X})}{\sum_{i=1}^{N}{\left(X_{i}-\bar{X}\right)}^{2}},\;S_{0}=\overset{N}{\underset{i=1}{\sum }}\overset{N}{\underset{j=1}{\sum }}W(i,\;j)$$ (4)

Based on the geographic distance spatial weights, we examine the spatial autocorrelation characteristics of carbon emissions from civil buildings in Chinese cities. Table 2 reports the results. From the results of the spatial autocorrelation test of total civil building carbon emissions and per capita civil building carbon emissions from 2010 to 2018, the Moran index was positive in all years, and all passed the significance test at the 1% level. This shows that the spatial distribution of carbon emissions from civil buildings in Chinese cities is not completely random, but has a significant positive spatial autocorrelation. That is, cities with higher carbon emissions from civil buildings tend to be adjacent to cities with higher carbon emissions. As a result, the phenomenon of spatial agglomeration between similar values appears. During the sample period, this spatial agglomeration phenomenon has shown an increasing trend. The Moran index based on the total carbon emissions from civil buildings has risen from 0.193 (2010) to 0.238 (2018), with an average annual increase of 2.66%. The Moran index based on the per capita values has changed more volatility, showing a fluctuating upward trend during the sample period, with an average annual growth rate of 3.49%. It can be seen that the trend of cities with similar civil building carbon emissions to be concentrated in space is becoming more and more obvious.

**Table 2:** Spatial autocorrelation test of carbon emissions from civil buildings.

Year	Total.			Per capita.		
	Moran index	z value	p value	Moran index	z value	p value
2010	0.193	5.008	0.00	0.212	4.514	0.00
2011	0.202	5.111	0.00	0.249	5.192	0.00
2012	0.194	4.905	0.00	0.259	5.378	0.00
2013	0.206	5.049	0.00	0.315	6.561	0.00
2014	0.206	5.046	0.00	0.302	6.297	0.00
2015	0.207	5.003	0.00	0.279	5.919	0.00
2016	0.207	5.029	0.00	0.264	5.619	0.00
2017	0.233	5.265	0.00	0.321	6.668	0.00
2018	0.238	5.231	0.00	0.279	5.829	0.00

**Fig 9:**
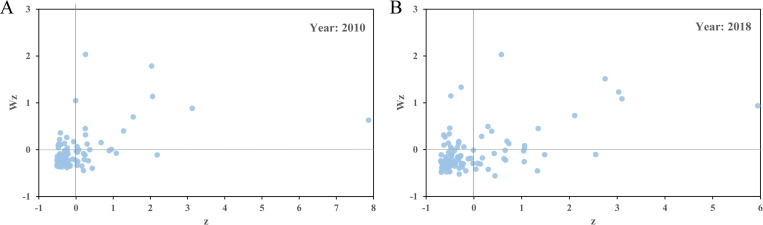
Moran scatter plot of total carbon emissions from civil buildings in Chinese cities in 2000 (A) and 2018 (B).

**Fig 10:**
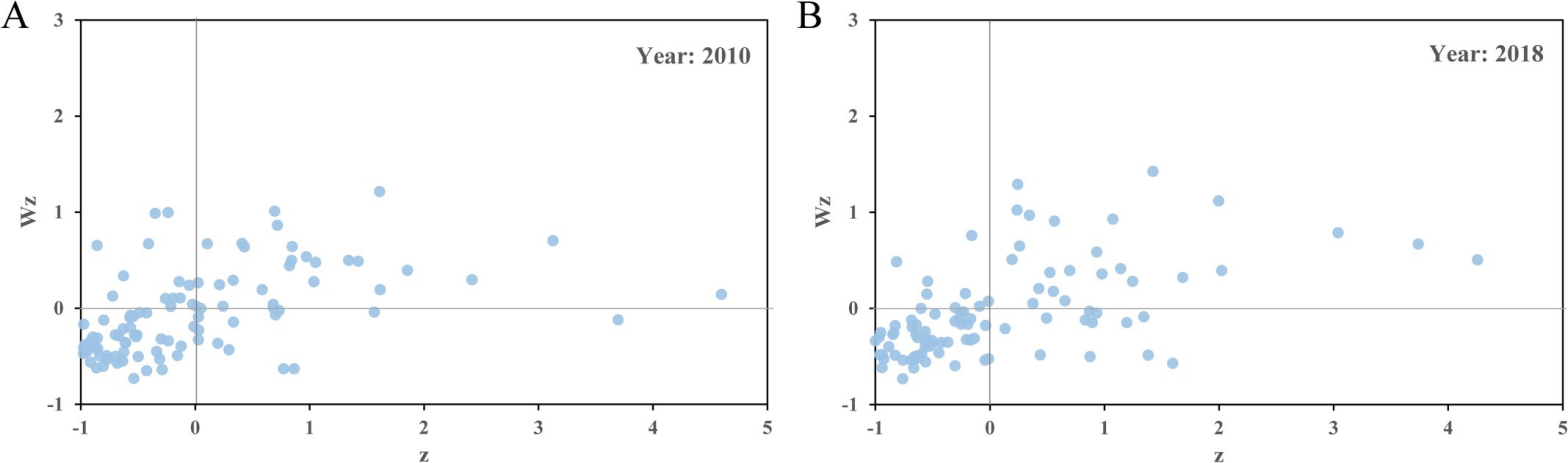
Moran scatter plot of per capita carbon emissions from civil buildings in Chinese cities in 2000 (A) and 2018 (B).

According to the Moran scatter plot of the total carbon emissions from civil buildings in Chinese cities (Fig 9), the "high-high" (HH) and "low-low" (LL) quadrants aggregate over 70% of the sample cities. "High-low" (HL) and "low-high" (LH) quadrants indicate a negative correlation in spatial distribution, where HL means a type of local high-value outlier, *that is*, the total carbon emission from civil buildings is relatively higher than that of the surrounding cities, and LH means a type of local low-value outlier, *that is*, the total carbon emission is relatively lower than those of surrounding cities. From the Moran scatter plot, the proportion of cities in these two quadrants is less than 30%. In general, it shows a strong positive spatial autocorrelation in the total carbon emissions from civil buildings in China. According to the Moran scatter plot of per capita carbon emissions from civil buildings (Fig 10), the number of cities in the HH and LL quadrants in 2010 accounted for 21.0% and 51.0% of the total number of sample cities, respectively. In 2018, the proportions of samples in the HH and LL quadrants were 23.1% and 59.6%, respectively. This indicates that the per capita carbon emissions from civil buildings also show a strong spatial agglomeration phenomenon.

### 4.2 Influencing factors of carbon emissions from civil buildings

In order to reveal the influencing factors of civil building carbon emissions, we use the static panel data model and the spatial dynamic panel data model, respectively. The latter model adds the time lag and the spatial lag of carbon emissions from civil buildings, while the former model does not. For the static panel data model, we choose the feasible generalized least squares method (FGLS) to estimate the parameters, so as to avoid the possible estimation errors caused by cross-sectional correlation, heteroscedasticity, and serial correlation. Regarding the parameter estimation of the spatial dynamic panel data model, since the model addition of the time lag and the spatial lag of the explained variable may cause endogenous problems, we choose to use the systematic GMM method to estimate the parameters. The system GMM method can more effectively use the information in the difference equation and the level equation, so the estimation results are more effective.

#### (1) Results of the static panel data model

In Table 3, models (1)—(3) report the FGLS estimation results of static panel data models with the total carbon emissions from civil buildings as the explained variable, and models (4)—(6) report the estimation results with per capita carbon emissions from civil buildings as the explained variable. For the estimation results with the total values as the explained variable, the estimated coefficients and the significance level of each variable are generally consistent across different models. The real GDP per capita, the level of urbanization, the total retail sales of consumer goods, the technical level, the fixed assets investment, marketization level, and regional dummy variables are significant at the 5% level. The estimation results of population density and industrial structure failed to pass the significance tests. For the estimation results with the per capita values as the explained variable, all the explanatory variables except the technical level are significant at the 5% level. In models (1) and (3), the linear terms of real GDP per capita and urbanization level are significantly positive, and the quadratic terms are significantly negative. This indicates a significant inverted U-shaped curve relationship between the total civil building carbon emissions and economic development. There is also an inverted U-shaped curve relationship between urbanization and carbon emissions from civil buildings.

**Table 3:** FGLS estimation results of the static panel data model.

Variables	Total.			Per capita.		
	(1)	(2)	(3)	(4)	(5)	(6)
Constant	-4.293***	-14.49***	-12.78***	-1.677***	-17.39***	-11.18***
Real GDP per capita	0.216***	0.046**	0.144***	1.192***	0.884***	1.168***
Quadratic of Real GDP per capita	-0.055***	-	-0.031***	-0.100***	-	-0.086***
Urbanization rate	0.386***	5.524***	4.651***	0.198***	8.106***	4.942***
Quadratic of urbanization rate	-	-0.631***	-0.527***	-	-0.963***	-0.582***
Population density	-0.001	0.011	0.005	-0.058***	-0.039**	-0.052***
Industrial structure	0.039	0.020	0.030	0.165***	0.131***	0.165***
Social consumption expenditure	0.468***	0.451***	0.462***	-0.110***	-0.151***	-0.126***
Technical level	-0.004	-0.009*	-0.010**	-0.003	-0.007	-0.007
Fixed assets investment	0.062**	0.061***	0.061***	0.021***	0.018***	0.017***
Marketization	0.014***	0.016***	0.016***	0.013**	0.015**	0.012*
Area	1.837***	1.919***	1.908***	1.023***	1.151***	1.111***
Wald	202018***	196807***	206887***	58346***	59165***	61699***

Note

***, **, * indicate the 1%, 5%, and 10% significance level respectively.

According to the estimation results of models (4)—(6), when the per capita values are used as the explained variable, the significant inverted U-shaped curve relationship still exists. Along with the development of the economy and urbanization in China, carbon emissions from civil buildings have gradually increased. After reaching the highest point, carbon emissions from civil buildings will decrease as the economy and urbanization develop further. Based on the emission data, the carbon emissions from civil buildings in most sample cities are still growing. It shows that most Chinese cities are mainly located on the left side of the inverted U-shaped curve. The carbon emissions from civil buildings will continue to be positively correlated with economic growth and urbanization in the future, demonstrating the urgency and importance of achieving energy conservation and emission reduction in civil buildings.

#### (2) Results of the spatial dynamic panel data model

Due to the temporal trend and spatial effect of urban carbon emissions, the current value of carbon emissions is affected by the historical values and the neighboring areas’ spatial effects. Therefore, we will use the spatial dynamic panel data model for the empirical analysis. First, we use the robust LM tests to identify the spatial lag model or the spatial error model is more suitable for analyzing the influencing factors of carbon emissions from urban civil buildings. Table 4 reports the results of LM tests.

**Table 4:** Results of LM tests for the spatial panel data.

LM Tests	Total.		Per capita.	
	*χ* ^2^	*p* value	*χ* ^2^	*p* value
no lag	142.116	0.000	128.573	0.000
no lag (robust)	101.377	0.000	111.351	0.000
no error	45.810	0.000	29.116	0.000
no error (robust)	5.071	0.024	11.894	0.001

Regardless of whether the total values or the per capita values are used as the explained variable, the results of the robust LM tests for the spatial lag model are significantly higher than those of the spatial error model. Therefore, we choose the spatial dynamic panel data lag model. Due to the addition of time lag and spatial lag of the dependent variable, the results of traditional estimation methods will be biased. We then use the systematic GMM estimation method (Sys-GMM) for the coefficient estimation of the spatial dynamic panel model. The system GMM estimation can automatically identify reasonable instrumental variables based on the time trend of the variables, making the estimation results more effective than traditional methods.

Table 5 reports the estimation results based on QMLE and Sys-GMM. Comparing the two estimation results, we see that the significance level of the Sys-GMM estimation coefficients is higher than the QMLE estimation result, indicating that the endogeneity problem has a relatively strong influence on the estimation result of the spatial dynamic panel data model. The Hansen-J statistics are not significant, reflecting that the choice of instrumental variables of the Sys-GMM model is appropriate. The AR (1) and AR (2) tests show that although the second-order difference of residuals is serially correlated, the first-order difference does not have serial correlation problems. Thus the original residual sequence is also not correlated. The estimation results of the above three types of statistics prove that the setting of the model is appropriate, and there is no bias due to the choice of instrumental variables. Based on the above discussion, we will focus on analyzing the Sys-GMM estimation results.

**Table 5:** Estimation results of spatial dynamic panel data.

Variables	Total.		Per capita.	
	QMLE	Sys-GMM	QMLE	Sys-GMM
Time lag of dependent variable	0.634***	0.845***	0.568***	0.657***
Real GDP per capita	0.103	0.024	0.604***	0.187***
Quadratic of Real GDP per capita	-0.038*	-0.020***	-0.070***	-0.011**
Urbanization rate	-1.369	0.542***	-1.907	0.049
Quadratic of urbanization rate	0.179	-0.047**	0.254	-0.055
Population density	-0.007	-0.102***	-0.177***	-0.205***
Industrial structure	0.000	-0.257***	0.279***	0.204***
Social consumption expenditure	0.104**	0.198***	-0.157***	-0.008
Technical level	-0.017*	-0.048***	-0.018*	-0.010***
Fixed assets investment	0.022**	0.004*	-0.012	-0.013***
Marketization	0.003	0.041***	0.019	0.034***
*ρ*	0.219***	0.068***	0.177***	0.118***
Hanson J		92.75		100.43
A-B AR(1)		-2.29**		-3.08**
A-B AR(2)		0.42		1.16

Note

***, **, * indicate the 1%, 5%, and 10% significance level respectively.

According to the estimation results of Sys-GMM in Table 5, the coefficient of the time lag of the dependent variable is positive and significant at 1%, indicating that the evolution of carbon emissions from urban residential buildings in China has a significant path dependence. The historical values will have a certain impact on the current carbon emissions from civil buildings. If the total values of the time lag term rise by 1%, the current total emissions rise by 0.845%; if the per capita values of the time lag term rise by 1%, the current per capita emissions rise by 0.657%. For the spatial effects, the estimation coefficient ρ of the spatial lag term is highly significant at both the total and per capita levels, and the coefficient values are all positive. It shows that the carbon emissions from civil buildings have significant spatial agglomeration characteristics. The increase in carbon emissions from neighboring areas will contribute to the increase in carbon emissions from local civil buildings.

After adding time lag term and spatial lag term to the models, there is still a significant inverted U-curve relationship between the total carbon emissions from civil buildings and economic development. The above results illustrate the urgency and importance of reducing carbon emissions in urban civil buildings. Due to the status quo of urban development, ensuring economic growth and urban development is still the main task of most Chinese local governments. As a result, most Chinese cities are still located in the rising part of the inverted U-shaped curve, and the civil building carbon emissions are still not "decoupled" from economic growth and urbanization. China’s urban development should accelerate its green and sustainable transformation by improving the efficiency of urban infrastructure and the green awareness of urban residents, so as to reduce carbon emissions from civil buildings while maintaining economic growth and urban development.

According to the estimation results of other explanatory variables, population density, technological progress, and fixed asset investment have significant negative effects on carbon emissions from civil buildings. Technological progress can improve the efficiency of energy use and thus reduce carbon emissions. The increase in urban population density will generate agglomeration effects and scale effects, which improve technology level, urban infrastructure use efficiency, energy use efficiency, and reduce the cost of carbon reduction facilities. Investment in fixed assets can replace outdated production technologies and increase the proportion of green technologies, which can significantly contribute to reducing carbon emissions from civil buildings. The above results show that structural reform on the production side, which leads the investment and technology progress in the direction of green and low-carbon, can significantly reduce carbon emissions. According to the estimation coefficients of industrial structure, the current adjustment of industrial structure in Chinese cities has been able to produce a negative effect on the total carbon emissions from civil buildings. This indicates that an effective carbon reduction mechanism for civil buildings is gradually being established on the production side. However, due to the extensive economic development, the optimization of industrial structure in China has not yet been able to effectively reduce carbon emissions from civil buildings at the per capita level.

The coefficient of marketization is positive with a high significance level based on the Sys-GMM estimation result. The coefficient of social consumption expenditure is significantly positive based on the estimation result of total values. This indicates that an effective carbon emission reduction mechanism for civil buildings has not yet been formed on the consumption side. Chinese urban residents have not yet established sufficient green awareness in consumption expenditure to promote the sustainable reduction of carbon emissions from civil buildings. The awareness of low-carbon consumption and environmental protection among urban residents should be vigorously promoted and raised. Realizing the transformation of consumption structure and lifestyle towards low carbon can effectively reduce energy consumption and carbon emissions during the operation of civil buildings.

## 5 Conclusions and policy implications

### 5.1 Conclusions

Along with urbanization, the proportion of carbon emissions from civil buildings in the total carbon emissions is gradually increasing. Reducing energy consumption and carbon emissions in the operational stage of civil buildings has become an effective means for China to achieve the "dual carbon" goal. Based on the data about carbon emissions from civil buildings in China, we examine the spatiotemporal characteristics of the civil building carbon emissions from the perspectives of temporal evolution trend, spatial distribution and its dynamic evolution, spatial difference and its decomposition, and spatial autocorrelation characteristics. Finally, we reveal the influencing factors of the carbon emissions from civil buildings using static panel data models and spatial dynamic panel data models. The study provides the following main conclusions: First, the carbon emissions from civil buildings in China have increased year by year during the sample period and have become an important source of the total carbon emissions. Second, there is a significant non-linear inverted U-shaped relationship between urban development and carbon emissions from civil buildings. The significant inverted U-shaped relationship still exists after adding the one-period lagged term of carbon emission indicators to the model. In other words, along with the development of cities in China, carbon emissions from civil buildings gradually increase. After reaching the peak, carbon emissions from civil buildings turn to decrease. Third, the estimation results of other explanatory variables show that population density, technological progress, and fixed asset investment negatively affect carbon emissions from civil buildings. Social consumption expenditure and the level of marketization positively affect the carbon emissions from civil buildings to some extent.

### 5.2 Policy implications

The critical policy implications for China’s reduction of carbon emissions from civil buildings and the overall realization of carbon emission reduction targets are indicated below:

Firstly, carbon emissions from civil buildings have become an important source of the total carbon emissions in China. Improving energy use efficiency in the operation of civil buildings is crucial to the achievement of China’s "dual carbon" goal. Therefore, in the process of formulating and implementing the policies for carbon emission reductions, it is necessary to strengthen the reduction of carbon emissions from civil buildings. At the same time, the spatial differences in carbon emissions should also be taken into consideration. A targeted total control system for the carbon emissions of civil buildings in various regions should be established to achieve differentiated treatment of carbon emission reduction policies. Specifically, it is necessary to reduce carbon emissions from civil buildings according to local conditions, and it also needs strengthen synergies between cities. According to the study in the paper, there is a certain amount of spatial difference in the distribution of China’s civil building carbon emissions. So, the responsibility for carbon reduction should vary from region to region, and the targets of reducing carbon emissions from civil buildings vary from city to city. Therefore, targeted control measures should be formulated according to the actual situation of each city. On the other hand, cities can learn from each other in civil building carbon reduction and actively absorb advanced control measures. This will strengthen the synergy and communication between cities and enable them to use various carbon reduction measures efficiently.

Secondly, the empirical results show that there is a significantly non-linear inverted U-shaped relationship between urbanization and carbon emissions from civil buildings, and the non-linear relationship is influenced by factors such as technology and industrial structure. At present, the urbanization rate of developed coastal cities has stabilized. However, the urbanization rate of small and medium-sized cities still has much room for improvement. Along with the urbanization of these cities, carbon emissions from civil buildings will continue to increase. During rapid urbanization, energy consumption and carbon emissions will increase significantly, which will increase the pressure on the environment. Therefore, China should abandon the traditional urbanization development model and actively expand the sustainable development approach of cities. The concept of low-carbon city development should be vigorously promoted in the process of urbanization. The use of new energy and clean production technologies should be actively incentivized through fiscal policies to increase the use of energy-efficient materials in civil buildings. Through the above methods, the marginal cost of urbanization can be reduced. Then, China as a whole will eventually enter the declining part of the inverted U-shaped curve, so as to realize the effective reduction of carbon emissions from civil buildings.

Thirdly, the carbon emissions from civil buildings can be significantly reduced through technological progress and industrial structure optimization on the production side. However, an effective carbon emission reduction mechanism for civil buildings has not yet been formed on the consumption side, which reduces the effectiveness of existing carbon emission reduction policies. Most urban residents in China have not yet established sufficient green awareness to promote a sustainable reduction of carbon emissions from civil buildings. There is still a need to improve the market-based mechanism to reduce carbon emissions from civil buildings through the change in income and energy consumption expenditure. In the process of formulating carbon emission reduction policies for civil buildings, the daily life of urban residents should be the focus of attention, and the energy consumption structure should be actively guided to change in the direction of low carbon, so as to realize the reduction of energy consumption and carbon emission during the operation of civil buildings.

Finally, technological progress is the main driving factor in reducing carbon emissions. The breadth and depth of application of green technology in the field of civil buildings should be further strengthened to fully exploit the potential of technological progress in energy saving and emission reduction. The key to achieving the "dual carbon" goal is replacing traditional technologies with green technologies. Enhancing the application of green technologies to achieve improved energy efficiency in the operation of civil buildings has become a necessary means of addressing the prominent energy consumption and carbon emission problems caused by rapid urbanization. From the perspective of policymakers, it is crucial to provide sufficient directional guidance and policy support for applying green technologies and energy-saving technologies to curb the growth of energy consumption and carbon emissions from civil buildings. Specifically, research incentive policies for civil building energy efficiency technologies should be actively established to achieve technological innovation in civil building. In addition, the transformation and promotion of energy-saving technologies in civil buildings should be accelerated, and the application of energy-saving materials and products in civil buildings should be promoted to enhance the effect of carbon emission reduction.

## Supporting information

S1 File Data. (CSV)

## References

[1] Sadorsky P. The effect of urbanization on CO_2_ emissions in emerging economies. Energy Economics. 2014; 41:147–153. doi: 10.1016/j.eneco.2013.11.007

[2] Yan Z, Xia L, Xiang W. Analyzing spatial patterns of urban carbon metabolism. Landscape & Urban Planning. 2014; 130:184–200. doi: 10.1016/j.landurbplan.2014.05.006

[3] Xu S, He Z, Long R, Shen W. Impacts of economic growth and urbanization on CO2 emissions: Regional differences in China based on panel estimation. Regional Environmental Change. 2016; 16(3): 777–787. doi: 10.1007/s10113-015-0795-0

[4] Wu Y, Shen J, Zhang X, Skitmore M, Lu W. The impact of urbanization on carbon emissions in developing countries: A Chinese study based on the U-Kaya method. Journal of Cleaner Production. 2017; 135: 589–603. doi: 10.1016/j.jclepro.2016.06.121

[5] Liu X, Bae J. Urbanization and industrialization impact of CO2 emissions in China. Journal of Cleaner Production. 2018; 172: 178–186. doi: 10.1016/j.jclepro.2017.10.156

[6] Wang Q, Su M. The effects of urbanization and industrialization on decoupling economic growth from carbon emission–A case study of China. Sustainable Cities and Society. 2019; 51:1–8. doi: 10.1016/j.scs.2019.101758

[7] Dong F, Wang Y, Su B, Hua Y, Zhang Y. The process of peak CO2 emissions in developed economies: A perspective of industrialization and urbanization. Resources, Conservation and Recycling. 2019; 141: 61–75. doi: 10.1016/j.resconrec.2018.10.010

[8] Wang Z, Cui C, Peng S. How do urbanization and consumption patterns affect carbon emissions in China—A decomposition analysis. Journal of Cleaner Production. 2019; 211: 1201–1208. doi: 10.1016/j.jclepro.2018.11.272

[9] Huo T, Li X, Cai W, Zuo J, Jia F, Wei H. Exploring the impact of urbanization on urban building carbon emissions in China: Evidence from a provincial panel data model. Sustainable Cities and Society. 2020; 56: 102068. doi: 10.1016/j.scs.2020.102068

[10] Lv Q, Liu H, Yang D, Liu H. Effects of urbanization on freight transport carbon emissions in China: common characteristics and regional disparity. Journal of Cleaner Production. 2019; 211: 481–489. doi: 10.1016/j.jclepro.2018.11.182

[11] Wang J, Song X, Chen, K. Which influencing factors cause CO_2_ emissions differences in China’s provincial construction industry: empirical analysis from a quantile regression model. Polish Journal of Environmental Studies. 2020; 29: 331–347. doi: 10.15244/pjoes/105239

[12] Liddle B, Lung S. Age-structure, urbanization, and climate change in developed countries: Revisiting STIRPAT for disaggregated population and consumption-related environmental impacts. Population & Environment. 2010; 31(5): 317–343. doi: 10.1007/s11111-010-0101-5

[13] Lin Q., Liu Y. China’s carbon dioxide emissions under the urbanization process: Influence factors and abatement policies. Economic Research Journal; 2010: 66–78 (in Chinese).

[14] Zhang C, Lin Y. Panel estimation for urbanization, energy consumption and CO2 emissions: a regional analysis in China. Energy Policy. 2012; 49: 488–498. doi: 10.1016/j.enpol.2012.06.048

[15] Afridi A, Kehelwalatenna S, Naseem I, Tahir M. Per capita income, trade openness, urbanization, energy consumption, and CO_2_ emissions: an empirical study on the SAARC Region. Environmental Science and Pollution Research. 2019; 26(29): 29978–29990. doi: 10.1007/s11356-019-06154-2 31414388

[16] Ali R, Bukhsh K, Yasin A. Impact of urbanization on CO2 emissions in emerging economy: Evidence from Pakistan. Sustainable Cities and Society. 2019; 48: 1–6. doi: 10.1016/j.scs.2019.101553

[17] He Z, Xu S, Shen W, Long R, Chen H. Impact of urbanization on energy related CO_2_ emission at different development levels: Regional difference in China based on panel estimation. Journal of Cleaner Production. 2017; 140: 1719–173. doi: 10.1016/j.jclepro.2016.08.155

[18] Fan Y, Liu L, Wu G, Wei Y. Analyzing impact factors of CO2 emissions using the STIRPAT model. Environmental impact assessment review. 2006; 26(4): 377–395. doi: 10.1016/j.eiar.2005.11.007

[19] Wang W, Kuang Y, Huang N, Zhao D. Empirical research on decoupling relationship between energy-related carbon emission and economic growth in Guangdong province based on extended Kaya identity. The Scientific World Journal. 2014; 2014:1–11. doi: 10.1155/2014/782750 24782666 PMC3981526

[20] Xu B, Lin B. How industrialization and urbanization process impacts on CO_2_ emissions in China: Evidence from nonparametric additive regression models. Energy Economics. 2015; 48:188–202. doi: 10.1016/j.eneco.2015.01.005

[21] Li K, Lin B. Impacts of urbanization and industrialization on energy consumption/CO_2_ emissions: Does the level of development matter. Renewable and Sustainable Energy Reviews. 2015; 52: 1107–1122. doi: 10.1016/j.rser.2015.07.185

[22] Huo T, Cao R, Du H, Zhang J, Cai W, Liu B. Nonlinear influence of urbanization on China’s urban residential building carbon emissions: New evidence from panel threshold model. Science of The Total Environment. 2021; 772(2):145058. doi: 10.1016/j.scitotenv.2021.145058 33770864

[23] Wang Q, Hang Y, Zhou P, Wang Y. Decoupling and attribution analysis of industrial carbon emissions in Taiwan. Energy. 2016; 113: 728–738. doi: 10.1016/j.energy.2016.07.108

[24] Xu X, Yang G, Tan Y, Zhuang Q, Tang X, Zhao K, et al. Factors influencing industrial carbon emissions and strategies for carbon mitigation in the Yangtze River Delta of China. Journal of Cleaner Production. 2017; 142: 3607–3616. doi: 10.1016/j.jclepro.2016.10.107

[25] Huang X, Xu X, Wang Q, Zhang L, Gao X, Chen L. Assessment of agricultural carbon emissions and their spatiotemporal changes in China, 1997–2016. International Journal of Environmental Research and Public Health. 2019; 16: 3105. doi: 10.3390/ijerph16173105 31455022 PMC6747386

[26] Prastiyo E, Hardyastuti S. How agriculture, manufacture, and urbanization induced carbon emission. Environmental Science and Pollution Research. 2020; 27(33): 42092–42103. doi: 10.1007/s11356-020-10148-w 32710357 PMC7654209

[27] Kang P, Song G, Chen D, Duan H, Zhong R. Characterizing the generation and spatial patterns of carbon emissions from urban express delivery service in China. Environmental Impact Assessment Review. 2020; 80: 106336. doi: 10.1016/j.eiar.2019.106336

[28] Hou H, Wang J, Yuan M, Liang S, Liu T, Wang H. Estimating the mitigation potential of the Chinese service sector using embodied carbon emissions accounting. Environmental Impact Assessment Review. 2021; 86:106510. doi: 10.1016/j.eiar.2020.106510

[29] Lu Y, Cui P, Li D. Carbon emissions and policies in China’s building and construction industry: Evidence from 1994 to 2012. Building and Environment. 2016; 95:94–103. doi: 10.1016/j.buildenv.2015.09.011

[30] Davis J, Peters P, Caldeira K. The supply chain of CO_2_ emissions. Proceedings of the National Academy of Sciences of the United States of America. 2011; 108(45): 18554–18559. doi: 10.1073/pnas.1107409108 22006314 PMC3215011

[31] Hou H, Bai H, Ji Y, Wang Y, Xu H. A historical time series for inter-industrial embodied carbon transfers within China. Journal of Cleaner Production. 2020; 264: 121738. doi: 10.1016/j.jclepro.2020.121738

[32] Hong J, Shen G, Guo S, Xue F, Zheng W. Energy use embodied in Chinas construction industry: A multi-regional input–output analysis. Renewable & Sustainable Energy Reviews. 2016; 53: 1303–1312. doi: 10.1016/j.rser.2015.09.068

[33] Fan J, Zhang Y, Wang B. The impact of urbanization on residential energy consumption in China: An aggregated and disaggregated analysis. Renewable & sustainable energy reviews. 2017; 75: 220–233. doi: 10.1016/j.rser.2016.10.066

[34] Yao X, Kou D, Shao S, Li X, Wang W, Zhang C. Can urbanization process and carbon emission abatement be harmonious. Environmental Impact Assessment Review. 2018; 71: 70–83. doi: 10.1016/j.eiar.2018.04.005

[35] Ehrlich R, Holdren P. Impact of Population Growth: Complacency concerning this component of man’s predicament is unjustified and counterproductive. Science. 1971; 171: 1212–1217. doi: 10.1126/science.171.3977.1212 5545198

[36] York R, Rosa E, Dietz T. STIRPAT, IPAT and ImPACT: analytic tools for unpacking the driving forces of environmental impacts. Ecological Economics. 2003; 46 (3): 351–365. doi: 10.1016/S0921-8009(03)00188-5

[37] Quah T. Empirics for growth and distribution: stratification, polarization, and convergence clubs. Journal of Economic Growth. 1997; 2(1): 27–59. doi: 10.1023/A:1009781613339.

[38] Chen Y. A tutorial on kernel density estimation and recent advances. Biostatistics & Epidemiology. 2017; 1(1): 161–187. doi: 10.1080/24709360.2017.1396742

[39] Decomposition Dagum C. and interpretation of Gini and the generalized entropy inequality measures. Statistica. 1997; 57(3): 295–308. doi: 10.6092/issn.1973-2201/1060

[40] Anselin L. The Moran scatterplot as an ESDA tool to assess local instability in spatial association. Spatial analytical perspectives on GIS. Routledge. 2019: 111–126.

